# Relationship between CCR and NT-proBNP in Chinese HF Patients, and Their Correlations with Severity of HF

**DOI:** 10.1155/2014/106252

**Published:** 2014-08-28

**Authors:** Zhigang Lu, Bo Wang, Yunliang Wang, Xueqing Qian, Wei Zheng, Meng Wei

**Affiliations:** ^1^Department of Cardiology, The 6th People's Hospital Affiliated to Shanghai Jiaotong University Medical College, No. 600 Yishan Road, Shanghai, China; ^2^Department of Clinical Laboratory, Dalian Municipal Central Hospital, Dalian, China; ^3^Department of Neurology, The 148th Hospital, Zibo, Shandong, China; ^4^Medlogic Healthcare Technology Company Ltd., Shanghai, China; ^5^Laboratory of Medicine, General Hospital of Shenyang Military Area Command, Shenyang, China

## Abstract

*Aim*. To evaluate the relationship between creatinine clearance rate (CCR) and the level of N-terminal pro-B-type natriuretic peptide (NT-proBNP) in heart failure (HF) patients and their correlations with HF severity. *Methods and Results*. Two hundred and one Chinese patients were grouped according to the New York Heart Association (NYHA) classification as NYHA 1-2 and 3-4 groups and 135 cases out of heart failure patients as control group. The following variables were compared among these three groups: age, sex, body mass index (BMI), smoking status, hypertension, diabetes, NT-proBNP, creatinine (Cr), uric acid (UA), left ventricular end-diastolic diameter (LVEDD), and CCR. The biomarkers of NT-proBNP, Cr, UA, LVEDD, and CCR varied significantly in the three groups, and these variables were positively correlated with the NHYA classification. The levels of NT-proBNP and CCR were closely related to the occurrence of HF and were independent risk factors for HF. At the same time, there was a significant negative correlation between the levels of NT-proBNP and CCR. The area under the receiver operating characteristic curve suggested that the NT-proBNP and CCR have high accuracy for diagnosis of HF and have clinical diagnostic value. *Conclusion*. NT-proBNP and CCR may be important biomarkers in evaluating the severity of HF.

## 1. Introduction

Chronic heart failure (CHF) is a disorder associated with high mortality and prolonged hospitalization; it affects more than 10 million people in the countries represented by the European Society of Cardiology [1]. With the development of the society and the increase in the aging population, the prevalence of hypertension, diabetes, and myocardial infarction (MI) is significantly higher than before, thus increasing the incidence of CHF [2]. Over the last decades, despite advances in treatment, the number of CHF deaths has increased steadily. Approximately 20% of deaths are reported per year due to CHF [3]. Heart failure not only declines heart pump function, but also produces the change of complex molecules, neuroendocrine, and inflammation immune system and makes many biomarkers at different stages including neurohormonal markers, inflammatory markers, markers of oxidative stress and myocardial injury, and remodeling markers. The assessment of these biomarkers, alone or in combination, may be useful in early diagnosis, differential diagnosis, prognosis, guiding treatment, and risk stratification for the heart failure patients. In recent years, with the emergence of the B-type natriuretic peptide (BNP) and clinical research, there is more focus on the development of biomarkers in heart failure [4].

The natriuretic peptide family mainly includes A-type natriuretic peptide (ANP) or atrial natriuretic peptide, B-type natriuretic peptide (BNP) or brain natriuretic peptide, C-type natriuretic peptide (CNP), renal natriuretic peptide, and Dendroaspis natriuretic peptide (DNP). The ANP and BNP are mainly secreted by the atrium and ventricle, respectively, and have similar effects, which are natriuretic and can inhibit renin angiotensin aldosterone system, and BNP is useful as a principal biomarker for CHF [5–9]. Since the level of BNP increases in heart failure, elevated plasma BNP concentration is used as a marker of heart failure. In recent years, like BNP, the NT-proBNP also was identified as a novel and important biomarker in heart failure to determine the severity of heart failure [10–12].

Heart failure and renal dysfunction are closely related. The ventricular dysfunction caused by CHF may lead to a series of adaptive responses, such as the activation of neuroendocrine system, peripheral vasoconstriction, and reduced renal perfusion pressure. All these changes can cause renal dysfunction, and the deterioration of renal function further increases the capacity of the load of the heart and leads to a vicious circle. The natriuretic peptide system can be activated in both heart failure and severe renal insufficiency patients. Renal dysfunction is often present in CHF patients with reported CCR lower than 60 mL/min in up to 50% of patients [13, 14].

Several studies have revealed that there is a relationship between NT-proBNP levels and clinical manifestations [4, 15, 16]. However, it remains unknown whether CCR and other biomarkers are correlated with the severity of heart failure. In this study, we aimed to determine whether the plasma levels of NT-proBNP, CCR, and other biomarkers altered with changes in the severity of heart failure and whether these markers are appropriate in immediately identifying symptomatic or asymptomatic heart failure in patients. While few similar studies have been conducted, this remains the first report in the Chinese population.

## 2. Materials and Methods

### 2.1. Ethics Statement

The investigation complied with the principles outlined in the Declaration of Helsinki [17]. The present cross-sectional study was performed in patients of outpatient setting. The study was approved by the Ethics Committee of the Hospital, Shanghai, Dalian, and Shenyang, China. Verbal informed consent was obtained from all patients. Each consent was recorded in the sample collection processing records and this consent procedure was approved by the Ethics Committee.

### 2.2. Patients

Three hundred thirty-six consecutive symptomatic or nonsymptomatic Chinese heart failure patients for suspected myocardial ischemia scheduled for coronary angiography were recruited between July 2011 and October 2012 at the 6th People's Hospital affiliated to Shanghai Jiaotong University Medical College, Dalian Municipal Central Hospital Affiliated of Dalian Medical University, and General Hospital of Shenyang Military Area Command, China. Severity of CHF was clinically evaluated according to the NYHA classification.

Two hundred and one Chinese patients were grouped according to the New York Heart Association (NYHA) classification as NYHA 1-2 and 3-4 groups and 135 cases out of heart failure patients as control group. Patients in NYHA class 1 showed cardiac disease but result in no limitation of physical activity. Ordinary physical activity does not cause undue fatigue, palpitation, dyspnea, or anginal pain. No cardiac disease, hypertension, or diabetes was diagnosed in control group.

A fasting venous blood sample was obtained for measurement of fasting glucose and HbA1c. Patients with HbA1c levels ≥6.5% were diagnosed as diabetic, even without previous history of diabetes. Body weight and height were measured to determine the body mass index (BMI; BMI, kg/m^2^ = weight (kg)/[height (m)]^2^) and blood pressure by standard methods.

### 2.3. Statistical Analysis

Data were expressed as mean ± standard deviation (SD) for continuous variables, or as percentages (%) for categorical variables. Statistical analysis was performed using SPSS for Windows (version 13.0). Variables such as age, sex, and smoking status were adjusted by covariance analysis. Repeated measures one-way ANOVA was used to determine the significance of trends within groups, and further comparison between the two groups was done using least significant difference procedure (LSD). Numerical data was compared using the Chi-square test. Spearman single-factor correlation analysis and Spearman coefficient of rank correlation were used to evaluate linear relationship between biomarkers and NYHA classification. Logistic regression was used to identify independent risk factors for heart failure. Association between variables in NT-proBNP and CCR was examined using Pearson's correlation coefficient and linear regression. Receiver operating characteristic (ROC) curves were used to obtain the biomarker cut-off points for predicting the prevalence of angiographic heart failure. The respective areas under the curve (AUC), sensitivity, and specificity were compared between biomarkers and NYHA classification. A value of *P* less than 0.05 was considered statistically significant.

### 2.4. Laboratory Assays

Fasting plasma glucose (FPG) was quantified by the glucose oxidase procedure and HbA1c was measured by ion-exchange high-performance liquid chromatography (HPLC, Bio-Rad, USA). Creatinine (Cr) and uric acid (UA) were measured by an enzymatic method with a chemical analyzer (Hitachi 7600-020, Tokyo, Japan); CCR was calculated using the Cockcroft-Gault formula. The chemiluminescence-based immunoanalytical system was used to determine plasma levels of NT-proBNP (VITROS 5600 integrated system, Johnson & Johnson Medical Company, USA).

### 2.5. Echocardiography

Two-dimensional and Doppler echocardiography scans were performed using the HP77020A echocardiograph (Hewlett Packard Company, USA) to assess the left ventricular end-diastolic diameter (LVEDD).

## 3. Results

### 3.1. Baseline Characteristics

The baseline characteristics of the patients are shown in Table 1. Of the 336 patients, 135 patients (40.18%, mean age = 65.84 ± 15.95 years, male = 47.40%) were in control group, 79 patients (23.51%, mean age = 69.91 ± 13.14 years, male = 54.43%) were in NYHA class 1-2, and 122 patients (36.31%, mean age = 68.27 ± 13.36 years, male = 63.93%) were in NYHA class 3-4.

There were no significant differences in age, BMI, and current smokers between any of heart failure groups and the control group (*P* > 0.05); the variables of male, hypertension, diabetes, NT-proBNP, CCR, Cr, UA, and LVEDD were significantly different among the three groups (all *P* < 0.01, excluded variable of male *P* < 0.05).

### 3.2. Comparison of NT-proBNP, CCR, Cr, UA, and LVEDD between the Heart Failure Groups and Control Group

The NT-proBNP, Cr, UA, and LVEDD levels were significantly higher in the NYHA class 1-2 and 3-4 groups than in the control group, and these variables in the NYHA class 3-4 group were significantly higher than that in the control and NYHA class 1-2 groups (all *P* < 0.01, excluded LVEDD level between NYHA class 1-2 and 3-4 groups, *P* < 0.05). The NT-proBNP level increased from control group (78.83 ± 15.27) to NYHA class 1-2 group (1611.54 ± 171.24) to class 3-4 group (3162.19 ± 453.21). The value for CCR significantly decreased from control group (89.94 ± 16.39) to NYHA class 1-2 group (59.43 ± 19.57) and class 3-4 group (53.57 ± 17.41) (*P* < 0.01). The patients with history of hypertension and diabetes were higher in the NYHA class 1-2 and 3-4 groups than in the control group (*P* < 0.05), and there was no significant difference between NYHA class 3-4 and 1-2 groups (Table 2).

### 3.3. Correlations Analysis of Individual Biomarkers with NYHA Classification in Heart Failure Groups

As shown in Table 3, the coefficient of rank correlation for NT-proBNP was 0.87, CCR was 0.74, Cr was 0.69, LVEDD was 0.44, and UA was 0.64, with *P* = 0.00. The variables of NT-proBNP, CCR, Cr, LVEDD, and UA showed positive correlation with the NHYA classification. With NHYA classification as dependent variable (*y* = 1, *n* = 0) and age, male, BMI, current smokers, hypertension, diabetes, NT-proBNP, CCR, Cr, UA, and LVEDD as independent variables, the results showed that NT-proBNP and CCR were independent risk factors for heart failure (Table 4).

The Pearson correlation analysis was carried out to determine the relationship between variables of NT-proBNP and CCR in control and heart failure groups.  Table 5 shows that there was a significant negative correlation between the levels of NT-proBNP and CCR (*r* = −0.62, *P* = 0.00).

In univariate linear regression analysis, CCR showed a significant negative correlation with NT-proBNP in the control and heart failure groups (*r* = −0.62, *P* = 0.00, Figure 2). This indicates that with the elevated NT-proBNP levels, CCR gradually reduced in the control group to NYHA class 1-2 to class 3-4 group.

### 3.4. Diagnostic Power of NT-proBNP and CCR for Heart Failure

**Figure 1:**
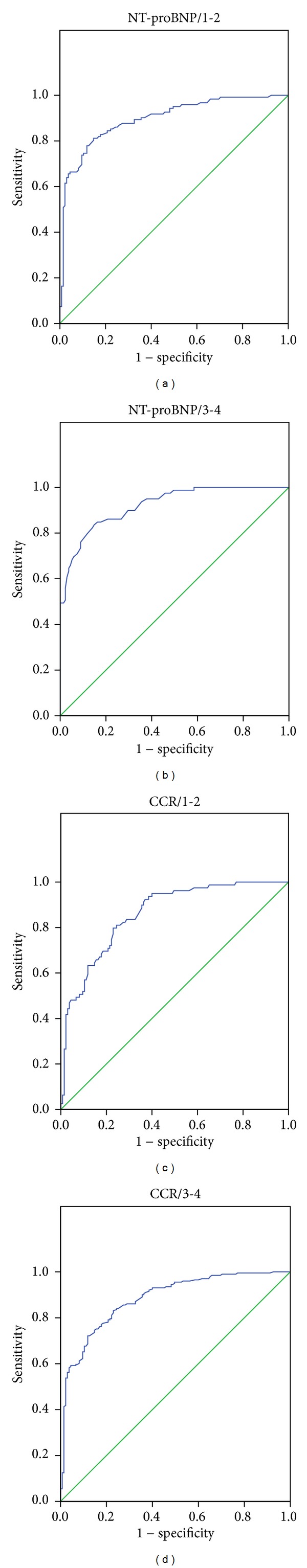
Receiver operating characteristic curve analysis of NT-proBNP and CCR for diagnosis for heart failure. ROC curve shows NT-proBNP for the prediction of heart failure patients with NHYA class: (a) 1-2 and (b) 3-4 groups; ROC curve shows CCR for the prediction of heart failure patients with NHYA class: (c) 1-2 and (d) 3-4 groups.

**Figure 2:**
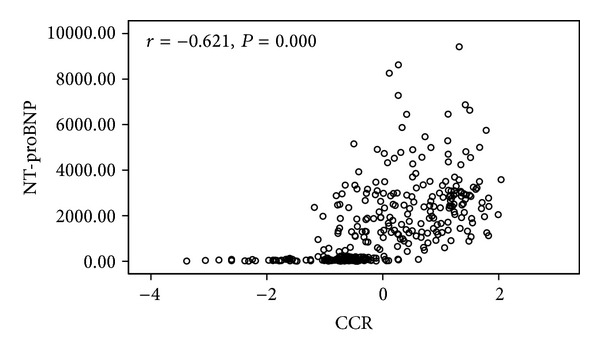
Linear regression analysis of the level of NT-proBNP with CCR (*r* = −0.621, *P* = 0.00, *n* = 336).

The ROC curves for NT-proBNP and CCR as indicators of heart failure are shown in Figure 1. The area under the ROC curve was higher for NT-proBNP (NHYA 1-2: 0.896; NHYA 3-4: 0.922) than for CCR (NHYA 1-2: 0.860; NHYA 3-4: 0.882). These results suggested that the NT-proBNP and CCR have high accuracy for diagnosis of heart failure and have clinical diagnostic value. The respective cut-off points for diagnosis of heart failure were estimated according to the ROC curves for NT-proBNP and CCR. With a cut-off value of 329.05 pg/mL, NT-proBNP had a sensitivity of 81.07% and a specificity of 75.62% for predicting NHYA 1-2, and a cut-off value of 324.40 pg/mL had a sensitivity of 82.34% and a specificity of 85.90% for predicting NHYA 3-4. Similarly, a cut-off value for CCR of 61.39 mL/min had a sensitivity of 74.71% and a specificity of 63.02% for predicting NHYA 1-2, and a cut-off value of 63.13 mL/min had a sensitivity of 82.42% and a specificity of 78.33% for predicting NHYA 3-4. Using these cut-off points, NT-proBNP showed higher sensitivity and specificity than CCR (Table 6).

## 4. Discussion

In this study, we observed that the variables, such as male, hypertension, diabetes, NT-pro BNP, CCR, Cr, UA, and LVEDD, were significantly different among all groups. Furthermore, we revealed that the biomarkers of NT-proBNP, Cr, UA, LVEDD, and CCR were positively correlated with the severity of heart failure.

The NT-proBNP level significantly increased and the value for CCR significantly decreased from control group to NYHA class 1-2 to 3-4 group. The levels of NT-proBNP and CCR were closely related to heart failure and were independent risk factors for patients with heart failure. At the same time, there was a significant negative correlation between the level of NT-proBNP and CCR. The area under the ROC curve suggested that the NT-proBNP and CCR have high accuracy in the diagnosis of heart failure with clinical diagnostic value.

Our findings are similar to the results of several previous studies where NT-proBNP plasma levels were closely related to the severity of heart failure [18]. Furthermore, in our study, the mean levels of NT-proBNP in the NYHA class 1-2 and 3-4 groups were greater than the cut-off points for diagnosis of heart failure, indicating that severity of heart failure increased gradually from control group to class 1-2 and 3-4 groups.

Heart failure and renal dysfunction often coexist as the visceral damage in one organ will result in the other organ's pathological changes accordingly. As two most important organs in the body, heart and kidney influence each other in the physiological and pathological processes, and the renal blood flow accounts for 20–25% of the total output of heart and plays an important role in regulating blood volume, blood vessels tension, and blood pressure change. In patients with heart failure, moderately elevated serum creatinine, without a history of chronic renal insufficiency, is often noticed. Therefore, our research focused on heart failure with no history of chronic kidney disease patients to observe the correlation between kidney index and cardiac function.

Determination of endogenous CCR can effectively evaluate the glomerular filtration function. The CCR can determine the degree of renal impairment and whether glomerular filtration function was damaged. Previous studies have shown that renal insufficiency is the risk factor for prognosis of patients with myocardial infarction, cardiac insufficiency, and hypertension [19–21].

Uric acid, a product of purine metabolism whose elevated concentration in CHF is a sign of damaged oxygen metabolism, is associated with the severity of cardiac dysfunction [22]. There is a correlation between uric acid and the existing cardiovascular disease risk factors such as hypertension, diabetes, hyperlipidemia, and obesity [23]. Increased blood UA levels in patients with CHF may be because of excretion of UA occurring mainly through kidneys, hypoxemia, increased anaerobic metabolism, and activation of xanthine oxidase. With the severity of heart failure, rate of anaerobic metabolism and quantity of lactic acid increase and the excretion of UA and lactate compete for the anion channel in the proximal convoluted tubule. The excretion of UA is reduced and results in the increased blood UA concentration. The cardiac output quantity is significantly reduced in the patients with heart failure, which results in decreased renal blood flow, damaged kidney, reduced glomerular filtration rate, decreased excretion of UA, and the increased level of UA [24].

Left ventricular end-diastolic diameter is used to determine the abnormal changes of cardiac systolic function. Cardiac ischemia causes the interruption of coronary blood flow and decline of myocardial contraction ability. Cardiac contraction ability is closely related to the size of myocardial ischemic area. When ischemic area size exceeds 15%, LVEDD value increases. There is evidence to suggest that NT-proBNP levels may reflect increased left ventricular wall stress in the absence of cardiac ischemia; thus, in this study we observed that the LVEDD increased from control to NYHA class 1-2 to 3-4 group, associated with heart failure severity.

## Figures and Tables

**Table 1:** Characteristics of the control, NYHA classification 1-2, and 3-4 groups.

Groups	Control (*n* = 135)	1-2 (*n* = 79)	3-4 (*n* = 122)	*F* value	*P* value
Age, year (mean ± SD)	65.84 ± 15.95	69.91 ± 13.14	68.27 ± 13.36	2.14	**0.12**
Male, % (*n*)	47.40 (64/135)	54.43 (43/79)	63.93 (78/122)	3.59	**0.03**
BMI, kg/m^2^ (mean ± SD)	23.48 ± 3.69	24.19 ± 4.31	24.05 ± 4.22	1.13	**0.33**
Current smokers, % (*n*)	27.40 (37/135)	29.11 (23/79)	17.21 (21/122)	0.55	**0.58**
Hypertension, % (*n*)	49.63 (67/135)	75.94 (60/79)	71.31 (87/122)	10.39	**0.00**
Diabetes, % (*n*)	16.30 (22/135)	30.38 (24/79)	38.52 (47/122)	8.43	**0.00**
NT-proBNP, pg/mL (mean ± SD)	78.83 ± 15.27	1611.54 ± 171.24	3162.19 ± 453.21	260.18	**0.00**
CCR, mL/min (mean ± SD)	89.94 ± 16.39	59.43 ± 19.57	53.57 ± 17.41	154.87	**0.00**
Cr, mg/dL (mean ± SD)	0.67 ± 0.15	1.07 ± 0.25	1.16 ± 0.23	195.55	**0.00**
UA, umol/L (mean ± SD)	299.22 ± 56.12	418.91 ± 49.21	471.54 ± 64.72	88.80	**0.00**
LVEDD, mm (mean ± SD)	45.21 ± 3.86	50.89 ± 6.65	52.85 ± 8.71	45.40	**0.00**

BMI: body mass index; CCR: creatinine clearance rate; Cr: creatinine; LVEDD: left ventricular end-diastolic diameter; NT-proBNP: N-terminal pro-B-type natriuretic peptide; NYHA: New York Heart Association; UA: uric acid.

**Table 2:** Paired comparison for biology markers between control group and heart failure group.

Variable	NYHA		SE	*P* value	95% CI	
					Lower	Upper
Male	0	2	0.07	0.32	−0.21	0.07
		3	0.06	0.01	−0.29	−0.04
	2	3	0.07	0.18	−2.24	0.05

Hypertension	0	2	0.07	0.00	−0.39	−0.13
		3	0.06	0.00	−0.33	−0.10
	2	3	0.07	0.49	−0.09	0.18

Diabetes	0	2	0.06	0.02	−0.26	−0.02
		3	0.05	0.00	−0.33	−0.11
	2	3	0.06	0.20	−0.21	0.04

NT-proBNP	0	2	153.31	0.00	−1834.29	−1231.12
		3	135.20	0.00	−3349.31	−2817.41
	2	3	156.30	0.00	−1858.11	−1243.20

CCR	0	2	2.49	0.00	25.62	35.40
		3	2.19	0.00	32.06	40.69
	2	3	2.53	0.02	0.88	10.85

Cr	0	2	0.03	0.00	−0.46	−0.35
		3	0.04	0.00	−0.54	−0.44
	2	3	0.03	0.01	−0.14	−0.02

UA	0	2	14.99	0.00	−149.18	−90.20
		3	13.22	0.00	−198.33	−146.31
	2	3	15.30	0.00	−82.70	−22.56

LVEDD	0	2	0.94	0.00	−7.52	−3.82
		3	0.83	0.00	−9.27	−6.01
	2	3	0.96	0.04	−3.85	−2.76

CCR: creatinine clearance rate; CI: confidence interval; Cr: creatinine; LVEDD: left ventricular end-diastolic diameter; NT-proBNP: N-terminal pro-B-type natriuretic peptide; NYHA: New York Heart Association; SE: standard error; UA: uric acid.

**Table 3:** Spearman correlation analysis of relations between variables and the NYHA classification (*n* = 336).

Variable	Correlation coefficient	Sig (2-tailed)
NT-proBNP	0.87	0.00
CCR	0.74	0.00
Cr	0.69	0.00
LVEDD	0.44	0.00
UA	0.64	0.00

CCR: creatinine clearance rate; Cr: creatinine; LVEDD: left ventricular end-diastolic diameter; NT-proBNP: N-terminal pro-B-type natriuretic peptide; UA: uric acid.

**Table 4:** Logistic regression analysis of risk factors for heart failure.

Variable	Regression coefficient	wald	*χ* ^2^ value	*P* value	Correct class
NT-proBNP	0.03	4.52	265.55	0.03	87.50%
CCR	20.82	6.08	441.48	0.01	99.70%

CCR: creatinine clearance rate; NT-proBNP: N-terminal pro-B-type natriuretic peptide.

**Table 5:** Pearson correlation analysis for NT-proBNP and CCR (*n* = 336).

Variable	NT-proBNP	CCR
NT-proBNP		
Correlation coefficient	1.00	−0.62
Sig (2-tailed)		0.00
CCR		
Correlation coefficient	−0.62	1.00
Sig (2-tailed)	0.00	

CCR: creatinine clearance rate; NT-proBNP: N-terminal pro-B-type natriuretic peptide.

**Table 6:** Cut-off points, sensitivity, specificity, and area under the curves for biomarkers and NYHA classification.

Marker	NYHA classification	Cut-off point	Sensitivity	Specificity	Area under ROC curve	SE	*P* value	95% CI	
								Lower	Upper
NT-proBNP	1-2	329.05 pg/mL	81.07%	75.62%	0.90	0.02	0.00	0.86	0.94
	3-4	324.40 pg/mL	82.34%	85.90%	0.92	0.02	0.00	0.89	0.96
CCR	1-2	61.39 mL/min	74.71%	63.02%	0.86	0.03	0.00	0.81	0.91
	3-4	63.13 mL/min	82.42%	78.33%	0.88	0.02	0.00	0.85	0.92

CCR: creatinine clearance rate; CI: confidence interval; NT-proBNP: N-terminal pro-B-type natriuretic peptide; NYHA: New York Heart Association; ROC: receiver operating characteristic; SE: standard error.
